# APOBEC3G encapsidation into HIV-1 virions: which RNA is it?

**DOI:** 10.1186/1742-4690-5-55

**Published:** 2008-07-02

**Authors:** Klaus Strebel, Mohammad A Khan

**Affiliations:** 1 Laboratory of Molecular Microbiology, National Institute of Allergy and Infectious Diseases, National Institutes of Health, 4/312, Bethesda, MD, 20892-0460, USA

## Abstract

APOBEC3G is a cytidine deaminase with potent antiviral activity. The protein deaminates single-stranded DNA but is known to bind cellular and viral RNAs. There is increasing evidence that RNA binding of APOBEC3G is important for packaging into viral particles. However, there is no consensus yet on the type of RNA involved.

## Commentary

**Figure 1:**
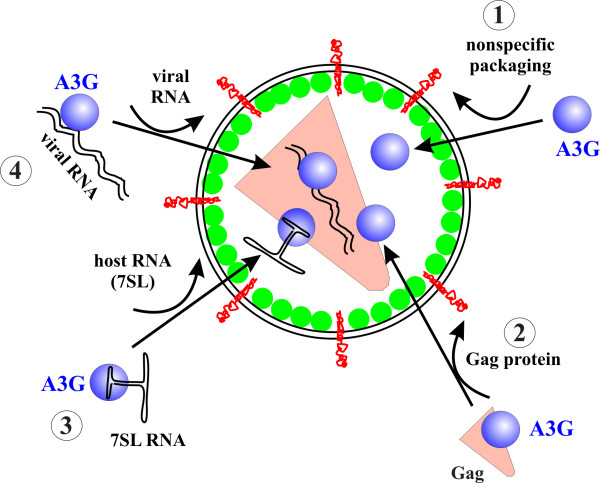
**Mechanism of APOBEC3G encapsidation**. Four different scenarios can be envisioned: (1) APOBEC3G is packaged non-specifically. This possibility seems unlikely given the known affinity of APOBEC3G to viral Gag proteins and viral and cellular RNAs. (2) APOBEC3G is packaged through interaction with Gag in an RNA-independent manner. Such a mechanism was initially proposed as discussed in the text. (3) APOBEC3G interacts with host RNA; in particular 7SL RNA was proposed to mediate encapsidation of APOBEC3G. (4) APOBEC3G is packaged through specific interaction with viral genomic RNA. This model is our favorite and is supported by the observation that point mutations in the 5' untranslated region of the viral genome can severely affect APOBEC3G packaging without affecting 7SL RNA encapsidation [6,10]. This model is also consistent with the data reported by Bach et al. [3].

APOBEC3G is a cytidine deaminase that was recently identified as the prime substrate of the HIV-1 viral infectivity factor Vif [1]. In the absence of Vif, APOBEC3G is packaged into viral cores and potently interferes with virus replication (for a recent review see [2]). While it is undisputed that much of APOBEC3G's antiviral activity comes from its encapsidation into virions, the mechanism of APOBEC3G packaging remains under discussion. Several studies conclude that APOBEC3G packaging is RNA independent and mediated by an APOBEC3G:Gag interaction while others identified a requirement for viral or cellular RNA in the APOBEC3G packaging process (Figure 1).

The study by Bach et al. [3] attempts to shed light onto the mechanism of APOBEC3G packaging. As far as the requirement of NC for packaging of 7SL RNA is concerned, the authors' data are in good agreement with a previous report by Wang et al. [4] showing that deletion of NC reduced packaging of 7SL RNA. Bach et al. disagree, however, with two other reports who found no significant NC-dependence for 7SL packaging [5,6]. Thus, the vote is tied at 2:2 on this issue. As far as the requirement of 7SL RNA for the packaging of APOBEC3G is concerned, Bach et al. fail to observe a correlation between APOBEC3G packaging and 7SL RNA incorporation. They therefore side with Khan et al. who previously also failed to see such a correlation [6]. Thus, the score is 2:1 against an involvement of 7SL RNA in the packaging of APOBEC3G. Unfortunately, Bach et al. did not analyze the importance of genomic RNA for APOBEC3G packaging, an issue on which Wang and Khan disagree. The score on that issue therefore remains tied at 1:1. Svarovskaia et al. also remain neutral on this issue by proposing that incorporation of APOBEC3G into HIV-1 virions involves both viral and nonviral RNAs [7].

There clearly is a trend in the field to accept the importance of RNA in the packaging of APOBEC3G into HIV-1 virions. But which RNA is it? There is no simple answer to this conundrum. Bach et al. point out that they and Wang et al. use a highly quantitative assay for determining encapsidation of 7SL RNA. So part of the difference may be attributed to differences in sensitivity. Some of the confusion may also come from the use of different experimental model systems, i.e. virus-like particles (VLP) versus whole virus. Also, it often is not appreciated that packaging of APOBEC3G into HIV virions or VLPs is as much a qualitative as it is a quantitative problem. For instance, the cytidine deaminase APOBEC3A is packaged into HIV virions but does not associate with viral cores and has no antiviral activity [8]. However, when the protein is artificially targeted to viral cores, APOBEC3A does develop antiviral properties [8,9]. Thus investigating APOBEC3G packaging into VLP rather than intact virions may allow the investigation of the quantitative aspect of APOBEC3G packaging but may fail to consider the qualitative aspect. Finally, protein overexpression is becoming an increasing concern in the APOBEC field. Some of the discrepant results on the role of RNA in the packaging of APOBEC3G into HIV-1 virions could potentially be explained by relative differences in protein expression.

In conclusion, the final verdict on what RNA guides the packaging of APOBEC3G into HIV-1 virions is still out and awaits further investigation. In particular, it remains to be determined whether APOBEC3G encapsidation entails a specific (e.g. viral RNA) and/or a non-specific component (e.g. cellular RNA). However, it is our (not completely unbiased) opinion that viral genomic RNA is excellently placed to make the finals.

## Competing interests

The authors declare that they have no competing interests.

## Authors' contributions

KS wrote the first draft and MAK made critical suggestions for improvement. All authors read and approved the final manuscript.
